# Short-term Forecasting of the Prevalence of Trachoma: Expert Opinion, Statistical Regression, versus Transmission Models

**DOI:** 10.1371/journal.pntd.0004000

**Published:** 2015-08-24

**Authors:** Fengchen Liu, Travis C. Porco, Abdou Amza, Boubacar Kadri, Baido Nassirou, Sheila K. West, Robin L. Bailey, Jeremy D. Keenan, Anthony W. Solomon, Paul M. Emerson, Manoj Gambhir, Thomas M. Lietman

**Affiliations:** 1 Francis I. Proctor Foundation, University of California San Francisco, San Francisco, California, United States of America; 2 Department of Ophthalmology, University of California San Francisco, San Francisco, California, United States of America; 3 Department of Epidemiology & Biostatistics, University of California San Francisco, San Francisco, California, United States of America; 4 Programme FSS/Université Abdou Moumouni de Niamey, Programme National de Santé Oculaire, Niamey, Niger; 5 Dana Center for Preventive Ophthalmology, Wilmer Eye Institute, Johns Hopkins University, Baltimore, Maryland, United States of America; 6 Clinical Research Unit, Department of Infectious and Tropical Diseases, London School of Hygiene & Tropical Medicine, London, United Kingdom; 7 Department of Control of Neglected Tropical Diseases, World Health Organization, Geneva, Switzerland; 8 International Trachoma Initiative, Atlanta, Georgia, United States of America; 9 Department of Epidemiology and Preventive Medicine, Monash University, Melbourne, Australia; Division of Global Health, UNITED REPUBLIC OF TANZANIA

## Abstract

**Background:**

Trachoma programs rely on guidelines made in large part using expert opinion of what will happen with and without intervention. Large community-randomized trials offer an opportunity to actually compare forecasting methods in a masked fashion.

**Methods:**

The Program for the Rapid Elimination of Trachoma trials estimated longitudinal prevalence of ocular chlamydial infection from 24 communities treated annually with mass azithromycin. Given antibiotic coverage and biannual assessments from baseline through 30 months, forecasts of the prevalence of infection in each of the 24 communities at 36 months were made by three methods: the sum of 15 experts’ opinion, statistical regression of the square-root-transformed prevalence, and a stochastic hidden Markov model of infection transmission (Susceptible-Infectious-Susceptible, or SIS model). All forecasters were masked to the 36-month results and to the other forecasts. Forecasts of the 24 communities were scored by the likelihood of the observed results and compared using Wilcoxon’s signed-rank statistic.

**Findings:**

Regression and SIS hidden Markov models had significantly better likelihood than community expert opinion (*p* = 0.004 and *p* = 0.01, respectively). All forecasts scored better when perturbed to decrease Fisher’s information. Each individual expert’s forecast was poorer than the sum of experts.

**Interpretation:**

Regression and SIS models performed significantly better than expert opinion, although all forecasts were overly confident. Further model refinements may score better, although would need to be tested and compared in new masked studies. Construction of guidelines that rely on forecasting future prevalence could consider use of mathematical and statistical models.

## Author Summary

Forecasts of infectious diseases are rarely made in a falsifiable manner. Trachoma trials offer an opportunity to actually compare forecasting methods in a masked fashion. The World Health Organization recommends at least three annual antibiotic mass drug administrations where the prevalence of trachoma is greater than 10% in children aged 1–9 years, with coverage at least at 80%. The Program for the Rapid Elimination of Trachoma trials estimated longitudinal prevalence of ocular chlamydial infection from 24 communities treated annually with mass azithromycin. Here, we compared forecasts of the prevalence of infection in each of the 24 communities at 36 months (given antibiotic coverage and biannual assessments from baseline through 30 months, and masked to the 36-month assessments) made by experts, statistical regression, and a transmission model. The transmission model was better than regression, with both far better than experts’ opinion. Construction of guidelines that rely on forecasting future prevalence could consider use of mathematical and statistical models.

**Trial Registration:**

Clinicaltrials.gov NCT00792922

## Introduction

The World Health Organization (WHO), the International Trachoma Initiative, Ministries of Health, and their partners aim to control blinding trachoma by 2020, implementing surgical campaigns, antibiotic distributions, hygiene initiatives, and environmental improvements [1]. Trachoma control is a massive undertaking: 50 million doses of antibiotics are now distributed annually, in 30 countries [2]. The Global Trachoma Mapping Project alone will complete population-based surveys in more than 1400 districts worldwide by the end of 2015 [3, 4]. Surveys and treatment histories are now available for the vast majority of trachoma-endemic districts worldwide [5]. However, we do not know where WHO goals will likely be and not be achieved. Decisions on when to start and stop treatments are still based on guidelines dependent in large part on expert opinion [6].

Mathematical models have provided insight into the transmission of infectious diseases including trachoma [7–15]. However, they have rarely been used to make *falsifiable* predictions. As a candidate for prediction, trachoma may have some advantages over other infectious diseases. Trachoma has no nonhuman reservoirs, no long-lasting latent stage, and as yet no clinically important drug resistance, simplifying modeling greatly compared to diseases such as cholera, onchocerciasis, and tuberculosis [1]. With many infectious diseases such as SARS and Ebola [16, 17], epidemics occur sporadically in time and place; forecasting can be made in a predictable time frame with post-treatment trachoma. Community-randomized trials have provided longitudinal assessment of multiple communities after mass antibiotic distributions. In a sense, after each mass treatment has brought infection to a low level, infection returns in a synchronized manner in a number of communities, offering results somewhat analogous to a repeated experiment.

Accurate forecasts could inform stakeholders of realistic goals, define trouble spots to focus resources, and suggest areas headed towards control even in the absence of intervention. Prediction has scientific value as well. The ability to predict the prevalence of an infectious disease is a test of our understanding of the epidemiology. Here, we use recent clinical trial data to forecast the prevalence of ocular chlamydial infection in children in 24 endemic communities in Niger. We compare model forecasts to expert opinions, and to a statistical regression that uses no special knowledge of the infectious process.

## Methods

### Data collection

Forty-eight communities were followed as part of the Niger arm of the Partnership for the Rapid Elimination of Trachoma (PRET) study. Communities were randomized to either mass antibiotics of the entire community, or antibiotics targeted just to children 12 years and younger. The 24 communities included in this study received annual antibiotic treatment of all ages. Communities were assessed at baseline and then biannually for 3 years. All individuals were offered antibiotic treatment annually, within two weeks of the assessment: children under 6 months, those allergic to macrolides, and pregnant women were offered topical tetracycline, and all others were offered a single dose of oral azithromycin (20 mg/kg for children and 1 gram for adults).

A random sample of 100 children 0–5 years old were selected from each community. If a community had less than 100 0–5 year-old children, then all were offered assessment. Each participating child had their upper right tarsal conjunctiva swabbed, and processed for PCR as previously described [18].

### Ethics statement

This study of de-identified data received ethical approval from the Committee on Human Research of the University of California San Francisco and was carried out in accordance with the Declaration of Helsinki. All adult subjects provided informed consent, and a parent or guardian of any child participant provided informed consent on their behalf. The informed consent given was oral: (a) we chose verbal consent because of the low literacy rates in the study area, (b) the IRB (10.00812) approved the use of oral consent, and (c) oral consent was documented on the registration form for each study participant prior to examination in the field.

### Survey methods

The WHO NTD-STAG Monitoring and Evaluation Working Group had a sub-group meeting to discuss trachoma surveillance on September 11–12, 2014 in Atlanta, GA, USA at the Task Force for Global Health, co-sponsored by WHO and the NTD Support Center. Fifteen trachoma experts were asked to forecast the 36 month prevalence of infection in the 24 communities of the PRET-Niger study described above, and were provided, for each community, the biannual prevalence estimates from 0–30 months, the antibiotic coverage at 0, 12, and 24 months, the estimated population of 0–5 years olds at baseline, and the number of children sampled at baseline (Table 1). For each of the 24 communities, the experts were asked to provide their median estimate at 36 months, as well as the lower and upper bounds of their centralized 95% credible interval (the 2.5^th^, 50^th^, and 97.5^th^ percentile of their belief). The *community* expert opinion was constructed by estimating each of the 15 individual’s distribution for each village (see Scoring below) and then taking the arithmetic average assuming equal weights, and was used as the primary survey forecast, although each individual’s forecast was also scored separately. The number of trachoma publications by each of the 15 experts was assessed by a PubMED (National Library of Medicine) search on December 1, 2014 (expert name as author AND “trachoma” as keyword).

**Table 1:** Format of forecast.

PRET-Niger forecasting exercise															
Village	Children 0–5 years		Antibiotic coverage*			Prevalence of infection in children 0–5 years by PCR						Your 36 month forecast			Observed
	Total	Tested	0	12	24	0*	6	12*	18	24*	30	Lower**	Median	Upper**	36
1	580	101	81%	81%	86%	30%	3%	3%	5%	6%	3%				12%
2	53	49	83%	89%	92%	8%	2%	2%	0%	0%	0%				0%
3	129	97	91%	79%	89%	8%	0%	0%	1%	4%	2%				2%
4	44	43	89%	91%	90%	51%	20%	14%	0%	0%	5%				23%
5	84	72	87%	86%	80%	25%	3%	3%	1%	3%	0%				0%
6	137	109	91%	90%	92%	13%	4%	2%	0%	0%	0%				0%
7	72	64	92%	88%	89%	2%	0%	0%	0%	0%	0%				0%
8	51	50	88%	84%	83%	20%	0%	3%	3%	3%	0%				0%
9	170	100	89%	81%	85%	3%	1%	1%	1%	1%	0%				0%
10	218	196	96%	84%	85%	28%	4%	6%	3%	2%	0%				2%
11	63	54	87%	88%	85%	7%	4%	6%	4%	2%	0%				0%
12	89	81	92%	87%	91%	48%	0%	0%	9%	25%	8%				16%
13	149	97	95%	82%	90%	3%	1%	3%	0%	1%	3%				5%
14	140	102	96%	92%	90%	39%	11%	13%	3%	5%	5%				8%
15	174	102	99%	92%	91%	31%	2%	6%	28%	28%	19%				11%
16	208	107	97%	96%	94%	35%	9%	20%	14%	15%	18%				22%
17	78	75	100%	95%	80%	9%	5%	4%	7%	13%	6%				13%
18	173	100	98%	94%	95%	58%	9%	11%	1%	2%	0%				0%
19	132	124	96%	97%	97%	27%	10%	7%	1%	5%	0%				0%
20	147	114	90%	97%	95%	24%	6%	16%	10%	18%	8%				5%
21	122	101	95%	94%	93%	11%	2%	2%	0%	0%	0%				0%
22	169	106	97%	95%	92%	17%	3%	1%	1%	5%	10%				13%
23	242	103	91%	94%	97%	5%	2%	1%	1%	0%	1%				0%
24	80	65	96%	82%	94%	6%	14%	4%	7%	2%	2%				7%
*j*	*N*_*j*_	*M*_*j*_	$c_{j}^{\left(1\right)}$	$c_{j}^{\left(2\right)}$	$c_{j}^{\left(3\right)}$	$S_{j}^{\left(0\right)}$	$S_{j}^{\left(1\right)}$	$S_{j}^{\left(2\right)}$	$S_{j}^{\left(3\right)}$	$S_{j}^{\left(4\right)}$	$S_{j}^{\left(5\right)}$				

*mass antibiotic distribution to all ages after sample collection at that time point

**lower and upper bounds of your 95% credible interval for the village

Forecasts by experts, regression, and SIS hidden Markov model were made using the data in this table, not including the observed 36 month results (right-hand column).

### Statistical methods

Linear mixed effects regression was used to model the prevalence at 12 months and 24 months based on observations at 6 months and at 18 months, respectively. A random intercept was used for each village. To improve normality and homoskedasticity, the square root transform was applied to the prevalence fractions. The fitted model was then used to predict the prevalence at time 36 based on observations at 30 months. Standard errors were obtained using clustered bootstrap. All calculations were conducted using *R* (*R* Foundation for Statistical Computing, Vienna, Austria, v.3.1 for Macintosh, package lme4). While the primary regression was of square-root transformed regression with a community-level random effect, we also included a linear regression model without a community-level random effect.

### Modeling methods

We constructed a stochastic transmission model of transmission of *Chlamydia trachomatis* infection over time. For village *j* (*j* = 1, … 24), we assumed a population of size *N*_*j*_, taken from the number of children aged 0–5 years found in the census at the time of treatment *k* (*k* = 1, 2, 3 corresponding to baseline, 12 and 24 months). We assumed a classical SIS (susceptible-infectious-susceptible) model structure, assuming that the force of infection is proportional to the prevalence of infection in the population of children aged 0–5 years with proportionality constant *β*, and a constant per-capita recovery rate *γ* [19]. Between periods of treatment, we assumed that the probability $p_{i,j}^{\left(k\right)}\;\left(t\right)$ that there are *i* infectives in village *j* at time *t* after treatment time point *k* obeys the following equations [20, 21]:

$$\frac{dp_{0,j}^{\left(k\right)}}{dt}=\gamma p_{1,j}^{\left(k\right)}$$

$$\frac{dp_{i,j}^{\left(k\right)}}{dt}=\beta \frac{\left(i-1\right)\left(N_{j}-i+1\right)}{N_{j}}p_{i-1,j}^{\left(k\right)}+\gamma \left(i+1\right)p_{i+1,j}^{\left(k\right)}-\beta \frac{i\left(N_{j}-i\right)}{N_{j}}p_{i,j}^{\left(k\right)}-\gamma ip_{i,j}^{\left(k\right)},\;\text{for}\;1\leq i\leq N_{j}-1$$ (1)

$$\frac{dp_{N_{j},j}^{\left(k\right)}}{dt}=\beta \frac{N_{j}-1}{N_{j}}p_{N_{j}-1,j}^{\left(k\right)}-\gamma N_{j}p_{N_{j},j}^{\left(k\right)}$$

To model treatment, we assumed that each child aged 0–5 years in village *j* has probability $c_{j}^{\left(k\right)}$ of receiving treatment with the antibiotic efficacy *e*_*k*_ for treatment period *k*. We modeled each treatment according to $p_{i,j}^{\left(k\right)}\;\left(t\;=\;0\right)\;=\;\sum_{i\prime =i}^{N_{j}}p_{i\prime ,j}^{\left(k,pre\right)}\;\left(\begin{array}{c} i\prime \\ i \end{array}\right)\;{\left(1-c_{j}^{\left(k\right)}\right)}^{i}{\left(c_{j}^{\left(k\right)}e_{k}\right)}^{i\prime -i}$, where *i*′ is the number of infected individuals of children aged 0–5 years eligible for treatment, $p_{i\prime ,j}^{\left(k,pre\right)}$ is the probability of *i*′ infected individuals of children aged 0–5 years before treatment time point *k*, and *i* is the number of infected individuals of children aged 0–5 years after treatment. Let $S_{j}^{\left(l\right)}$ and $M_{j}^{\left(l\right)}$ be the observed number of PCR-positive individuals of children aged 0–5 years and the sample size at each observation time point *l* (*l* = 0, 1, 2, 3, 4, and 5 corresponding to baseline, 6, 12, 18, 24 and 30 months, respectively) for village *j*, and *S*_*j*_ be the possible number (ranging from 0 to $M_{j}^{\left(l\right)}$) of positive individuals of children aged 0–5 years detected in the sample at observation time point *l*. From village *j* with population (children aged 0–5 years) size *N*_*j*_ of which the number *Y*_*j*_ of infectives equals *i*, the probability *P*(*S*_*j*_ = *s*|*Y*_*j*_ = *i*) that *s* positives are observed from a sample of size *M*_*j*_ is given by the hypergeometric distribution: $\left(\begin{array}{c} i\\ s \end{array}\right)\;\left(\begin{array}{c} N_{j}-i\\ M_{j}^{\left(l\right)}-s \end{array}\right)/\left(\begin{array}{c} N_{j}\\ M_{j}^{\left(l\right)} \end{array}\right)$. We assumed a standard beta-binomial prior (the binomial distribution in which the probability of success at each trial follows the beta distribution) $P\left(Y_{j}=y\right)=\left(\begin{array}{c} N_{j}\\ y \end{array}\right)\frac{\text{B}\left(y+\mu ,N_{j}-y+\rho \right)}{\text{B}\left(\mu ,\rho \right)}$ (where the shape parameters *μ* and *ρ* for each treatment were computed from the observed distribution of infection of 24 villages at baseline, 12 and 24 months, B(*z*_1_, *z*_2_) is the beta function) [22]. The pre-treatment prevalence distribution was then computed for each village by applying Bayes’ theorem:

$$p_{i,j}^{\left(k,pre\right)}=P\left(Y_{j}=i|S_{j}=s\right)=\frac{P\left(S_{j}=s|Y_{j}=i\right)P\left(Y_{j}=i\right)}{\sum_{i=0}^{N_{j}}P\left(S_{j}=s|Y_{j}=i\right)P\left(Y_{j}=i\right)}.$$ (2)

For each village *j*, the initial condition is determined from Eq (2), and the system numerically integrated for six or twelve months according to Eq (1). Specifically, for each village *j*, the pre-treatment distributions of *k*th treatment is $p_{i,j}^{\left(k,pre\right)}=P\left(Y_{j}=i|S_{j}=S_{j}^{\left(2k-2\right)}\right)$. Given the number *i* of infected individuals of children aged 0–5 years, we computed the probability of the observed data of treatment *k* in village *j* according to $P\left(S_{j}=s\right)=\sum_{i=s}^{N_{j}}p_{i,j}^{\left(k\right)}\left(\tau \right)\left(\begin{array}{c} i\\ s \end{array}\right)\left(\begin{array}{c} N_{j}-i\\ M_{j}-s \end{array}\right)/\left(\begin{array}{c} N_{j}\\ M_{j} \end{array}\right)$ (where *M*_*j*_ here denotes the sample size at one of the observation time points in the period *k*, and *τ* (6 or 12 months) is the interval between treatment time point and observation time point). We assumed independent villages, so that the total loglikelihood at time *τ* months after each treatment *k* may be computed by summing over all 24 villages $\sum_{j=1}^{24}\text{log}\left(\sum_{i=0}^{N_{j}}p_{i,j}^{\left(k\right)}\left(\tau \right)\left(\begin{array}{c} i\\ s \end{array}\right)\left(\begin{array}{c} N_{j}-i\\ M_{j}-s \end{array}\right)/\left(\begin{array}{c} N_{j}\\ M_{j} \end{array}\right)\right)$.

The transmission coefficient and antibiotic efficacy in the model were optimized by using the Metropolis algorithm with the total likelihood of three treatment periods to fit the model to the observed numbers of PCR-positive individuals of children aged 0–5 years in each village at 6, 12, 18, 24 and 30 months [23]. Forecasting the distribution of the observed number of PCR-positive individuals of children aged 0–5 years in a village at 36 months, conditionally on the observed numbers of PCR-positive individuals of children aged 0–5 years at baseline, 6, 12, 18, 24 and 30 months from the same village, was done by using a hidden Markov model according to the equation of forecast distribution [24].

The primary modeling forecast was pre-specified as the SIS process model with a random effect, although the SIS model without a random effect was included as a sensitivity analysis. In addition, the forecast of each model as a distribution over 101 discrete units was included as a comparison to the distribution estimated by minimizing the Fisher’s information (which allows a symmetric credible interval to approach a normal distribution, as well as the flexibility of asymmetric credible intervals to represent skewed distributions). Sensitivity analyses included changing the fixed mean infection duration assumed in the model to be 6 months, to 3 months or to 12 months.

### Scoring

To ensure a fair comparison, all forecasts were scored from the proposed median and 95% CrI. Given the denominator of the sample for each village at 36 months, the discrete distribution which minimized the Fisher’s information while constrained to that expert’s median and 95% CrI was estimated (*Mathematica 10*.*0*). As a sensitivity analysis, the SIS model forecasts were also presented as a distribution from 0 to 100%, with the score compared to the score derived from the median and 95% CrI. The modeler, statistician, and each of the 15 experts surveyed were all masked to the 36-month results, as well as to the forecasts made by others. Different forecasts were pairwise compared using Wilcoxon’s signed-rank test (*Mathematica 10*.*0*), using the Holm–Bonferroni multiple comparison correction, assuming 3 tests.

As a sensitivity analyses, we assessed whether the likelihood of the observed data would be greater (or lesser) had each forecast been more (or less) certain. Specifically, we perturbed each forecast by taking the density at each possible prevalence to the 1+*ϵ* power, normalizing, and determining the likelihood of the observed data. Note that this maintains the support of a forecast, maintains the ordering of the outcomes, and increases the Fisher’s information proportionally by ϵ (or decreases information proportionally for *ϵ*<0).

## Results

At the baseline census, communities had a mean of 146 children (95% CI 137 to 155) aged 0 to 5 years. The mean antibiotic coverage of children was 92.3% at baseline, 89.0% at 12 months, and 89.8% at 24 months. At baseline, the estimated prevalence of infection in the 24 communities ranged from 2% to 58% with a mean prevalence of 21.1% (95% CI 19.8% to 22.5%) [18]. The community prevalence of infection at each biannual visit is displayed in Table 1. The observed prevalence of infection at 36 month which was to be forecasted ranged from ranged from 0% to 22.5% with a mean prevalence of 5.8% (95% CI 5.2% to 6.4%).

**Fig 1:**
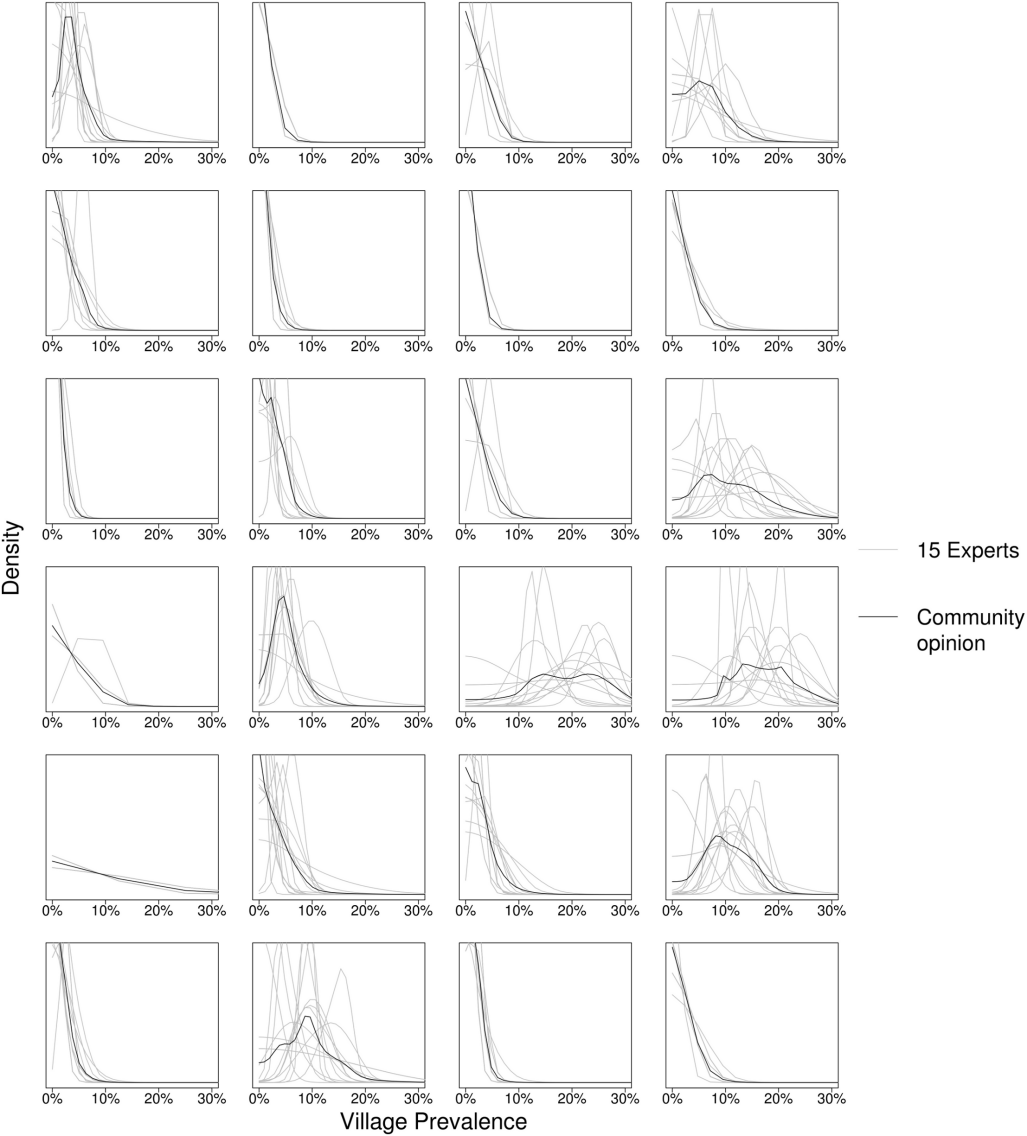
Survey results. 15 experts’ forecasts of the 36 month prevalence in each of 24 communities. Expert’s forecast distributions (grey curves) were estimated from their expected median and 95% CrI bounds for each community. Experts’ distributions could overlap when identical medians and bounds were submitted. The mean (black curve) is used to represent the community forecast.

**Fig 2:**
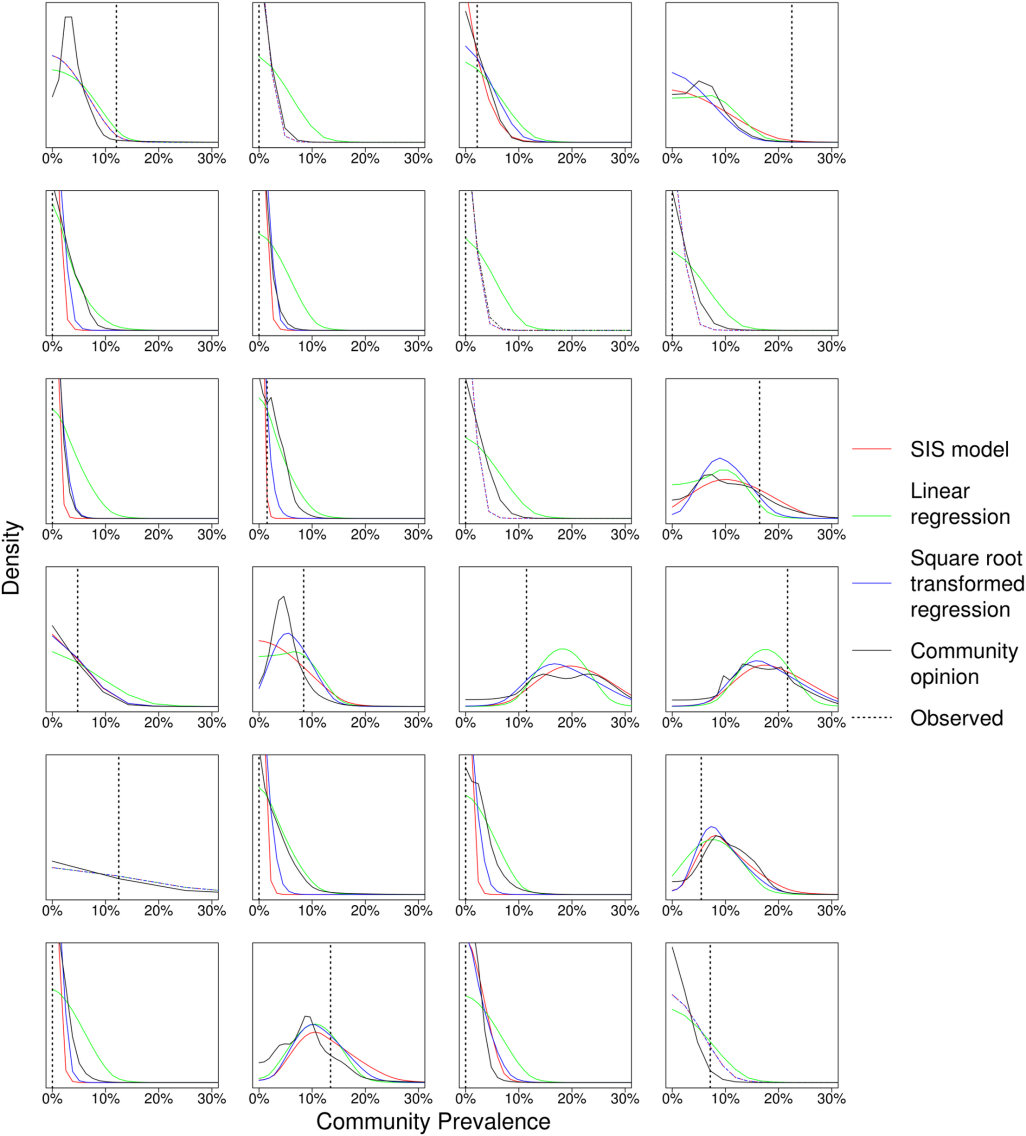
Different forecast methods versus observed result. Regressions (linear regression as green curve, square root-transformed blue), SIS hidden Markov Model (red), community of experts (black), and observed 36-month prevalence (dotted bar). Forecasts could overlap.

The 15 experts provided forecasts for each of the 24 communities, with the mean taken as the community forecast (Fig 1). Fig 2 shows the forecast distributions for the community of experts, regression, and the SIS model, and Table 2 ranks the likelihood of the observed 36-month prevalence for each (S1 Fig and S1 Table in Supporting Information show the difference between observed and forecast prevalence). The estimated parameters of the SIS model with random effect are shown in Table 3. The SIS model and the square root-transformed regression had significantly better likelihood than the experts (*p* = 0.004 and *p* = 0.01, respectively), and than the linear regression (*p* = 0.01 and *p* = 0.02, respectively). All forecasts were positively biased, on average estimating a greater prevalence than was observed. All forecasts had a lower (worse) likelihood if their Fisher’s information was marginally increased. No individual expert forecast was better than the community forecast (the mean of the 15 experts).

**Table 2:** Forecast scores and bias.

Model	log_e_ likelihood	Bias
**SIS hidden Markov model with random effect**	-41.03	+0.69
**SIS hidden Markov model without random effect**	-41.57	+0.64
**square root-transformed regression**	-42.90	+0.61
**community of experts**	-48.65	+1.75
**linear regression**	-51.88	+1.44
**individual experts, median result (range of *n* = 15)**	-61.07 (-53.84 to -104.95)	+0.90

Forecast were scored as the loglikelihood of observing the 24 community-level prevalence of ocular chlamydial infection at 36 months, with a higher (less negative) loglikelihood indicating a better forecast. Positive bias indicates that the expectations for the 24 communities were on average higher than the observed prevalence.

**Table 3:** Estimated parameters of the SIS model with random effect.

Duration of infection	Efficacy $\hat{e}$ (95% CI)	Mean of $\log_{e}\hat{\beta }$ (95% CI)	SD of $\log_{e}\hat{\beta }$ (95% CI)
**6-month**	0.836 (0.773, 0.886)	-1.403 (-1.529, -1.289)	0.035 (0.002, 0.098)
**3-month**	0.678 (0.561, 0.787)	-0.989(-1.373, -0.605)	0.033 (0.001, 0.092)
**12-month**	0.897 (0.853, 0.936)	-1.651 (-1.805, -1.519)	0.045 (0.002, 0.133)

Given a 6, 3, or 12months of infection duration, we estimated the overall efficacy, the mean and standard deviation of ${\text{log}}_{e}\;\hat{\beta }$ (assuming that the logarithm of transmission coefficient *β* is from a normal distribution) based on the observed data of 24 communities. Estimation was done by using MCMC.

A priori, the SIS model assumed a mean duration of infection of 6 months, obtaining a loglikelihood of the observed 36 month data of -41.03. Had we assumed the mean duration of infection was 3 months or 12 months, the loglikelihood would have been -41.47 or -39.91, respectively. If we had assumed the 6 month duration of infection, but did not use a community-level random effect, the likelihood score would have been -41.57. To fairly compare the different methods, the distribution of each forecast was estimated by minimizing the Fisher’s information given the estimated median and 95% CrI. For the SIS model, we also expressed each full distribution, obtaining a loglikelihood score of -40.90, or nearly the same as the -41.03 obtained from minimizing the Fisher’s information.

The mean number of trachoma citations on PubMED by the experts was 42 (range 0 to 133). The likelihood score and number of publications was actually inversely correlated (Spearman’s correlation -0.33, *p* = 0.24), thus we were unable to demonstrate that this measure of expertise was associated with better forecasting.

We performed logistic regression, assuming the individual PCR results most likely to have obtained the observed pooled results, but this performed no better than linear regression of the square-root transformed regression.

## Discussion

An SIS hidden Markov model and a regression model both produced forecasts with significantly higher likelihood of the observed data than a community of experts. The SIS model, which attempted to utilize an understanding of the infectious process and mass treatment, performed significantly better than linear regression, but only slightly (and not significantly) better than regression of the square root-transformed prevalence.

In general, more uncertainty resulted in better scoring forecasts. For every forecast a mathematical perturbation which reduced the Fisher’s information resulted in a higher likelihood of the observed data. The inclusion of a community-level random effect in the SIS hidden Markov model improved forecasting, perhaps by increasing uncertainty. The composite survey contained less information than any individual survey, and did better than any single individual forecast. The benefit of adding uncertainty could suggest that forecasts are inherently over-confident, or that additional variance components of the data were not considered by any of the methods.

Even though the SIS hidden Markov model and regression model had significantly higher likelihood than the community experts, the forecasted distributions of prevalence (as shown in Fig 2) by all models were very similar and did not show which model was significantly better than other models. With more available data, models could improve forecasting. The SIS hidden Markov model did not include infection from outside the population of children aged 0–5 years in each community. Our previous model [13] used a simple constant exogenous infection rate to represent infection from older children or adults to children aged 0–5 years within the same community, and did not find significant differences between the estimated transmission coefficients with and without the exogenous infection rate for different durations of infection. Of course, such models could be further refined to reflect age structured transmission dynamics. In this setting, the other age groups (older children and adults) were being treated as well, and other studies have shown consistently higher prevalence in small children than in other age groups (e.g. [25]).

The prevalence of infection in different communities is clearly correlated visit-to-visit, with visits 6-months apart having a higher correlation than visits further apart. However, there may be a fundamental limit to the predictability at the community level, simply due to the vagaries of who infects whom and when they do so. Mathematical models and cross-sectional empirical studies have suggested that as disease is disappearing, the prevalence of infection should form an exponential distribution (or its discrete analog, a geometric distribution), whether the disappearance is due to mass antibiotics, environmental improvements, or a secular trend [26, 27]. This exponential distribution has a much heavier tail than, for example, the normal distribution, so outliers are to be expected even when all communities are assumed to have identical transmission characteristics. Six-months is a relatively short period in trachoma control—programs typically reassess endemicity every 1–5 years. If predictability decreases as time increases between visits, than we would expect that apparent hotspots at one visit may not be the most affected areas at a subsequent visit. This has been termed *chasing ghosts* by trachoma programs (personal communication, PME).

Forecasts, whether made by experts, statistics, or mathematical transmission models, are rarely done in a falsifiable manner. Here, all participants were presented with identical information and masked to the results and to the other forecasters. Forecasts described the distribution of all possible outcomes, not a prediction of the single most favorable, and were scored in a pre-specified manner. The availability of results from 24 communities allowed a statistical comparison between forecasts, reducing the chance that the overall score would be dependent on a single fortunate guess.

Current WHO guidelines for starting mass drug administration are based on the district prevalence of the clinical signs of disease rather than infection, and future studies could assess forecasting at that level. In this study, we forecasted the community-level prevalence of ocular chlamydial infection. WHO guidelines currently include sub-district level intervention, at least for hypo-endemic districts with 5–10% prevalence of clinical activity in children. Individual community-level forecasting may become important for surveillance after mass antibiotic administrations have been discontinued.

Programs currently make decisions based on recommendations offered by the WHO [1]. Guidelines have relied heavily on extrapolation of existing evidence and expert opinion, since not all scenarios have been, or likely will ever be, tested in community-randomized trials. Forecasting at the individual community level has not been particularly successful. While forecasting at the district level may be more feasible than forecasting at the individual community level, statistical and transmission model forecasts should be evaluated. If proven more effective, as they were in this setting, then it may be reasonable for programmatic decisions to be based on statistical or modeling forecasts rather than just expert opinion.

## Supporting Information

S1 Fig Different forecast methods versus observed result. Regressions (linear regression, green; square root-transformed, blue), SIS hidden Markov Model (red), and community of experts (black), with mean (solid circle) and 95% CI (circle).
(TIFF)

S1 Table Difference between observed result and forecast by SIS, linear regression, square root transformed regression, and community opinion. (DOCX)
