# Correction: Short-term Forecasting of the Prevalence of Trachoma: Expert Opinion, Statistical Regression, versus Transmission Models

**DOI:** 10.1371/journal.pntd.0004129

**Published:** 2015-09-29

**Authors:** Fengchen Liu, Travis C. Porco, Abdou Amza, Boubacar Kadri, Baido Nassirou, Sheila K. West, Robin L. Bailey, Jeremy D. Keenan, Anthony W. Solomon, Paul M. Emerson, Manoj Gambhir, Thomas M. Lietman

There are errors in the Funding section. The correct funding information is as follows: This project was supported by Bill and Melinda Gates Foundation (grant number: 48027, grant recipient: TML, www.gatesfoundation.org), a Models of Infectious Disease Agent Study grant (grant number: U01GM08778, grant recipient: TCP, www.nigms.nih.gov/Research/SpecificAreas/MIDAS) from the National Institute of General Medical Sciences at the National Institutes of Health, and the NTD Modelling Consortium by the Bill and Melinda Gates Foundation in partnership with the Task Force for Global Health. The funders had no role in study design, data collection, data analysis, data interpretation, or writing of the article. The corresponding author had full access to all the data in the study and had final responsibility for the decision to submit for publication.

There is an omission in the Acknowledgments section. Please see the update Acknowledgments section here: In addition to the PRET, MIDAS and NTD Modelling Consortium study sponsors, the authors thank the data and safety monitoring committee, including Douglas Jabs, MD, MBA (chair), Antoinette Darville, MD, Maureen Maguire, PhD, and Grace Saguti, MD, who were generous with their time and advice and met before and during the study. The authors thank all of our colleagues in Niger at Programme National de Santé Oculaire who collected samples and data. We also thank the NTD support center, the Task Force for Global Health, and the 15 experts surveyed at the WHO NTD-STAG Monitoring and Evaluation Working Group meeting.
