# Supplementary figures and images for: Short-term Forecasting of the Prevalence of Trachoma: Expert Opinion, Statistical Regression, versus Transmission Models
Source: PLoS Negl Trop Dis. 2015 Aug 24;9(8):e0004000. doi: 10.1371/journal.pntd.0004000 (PMC4547743; doi:10.1371/journal.pntd.0004000)

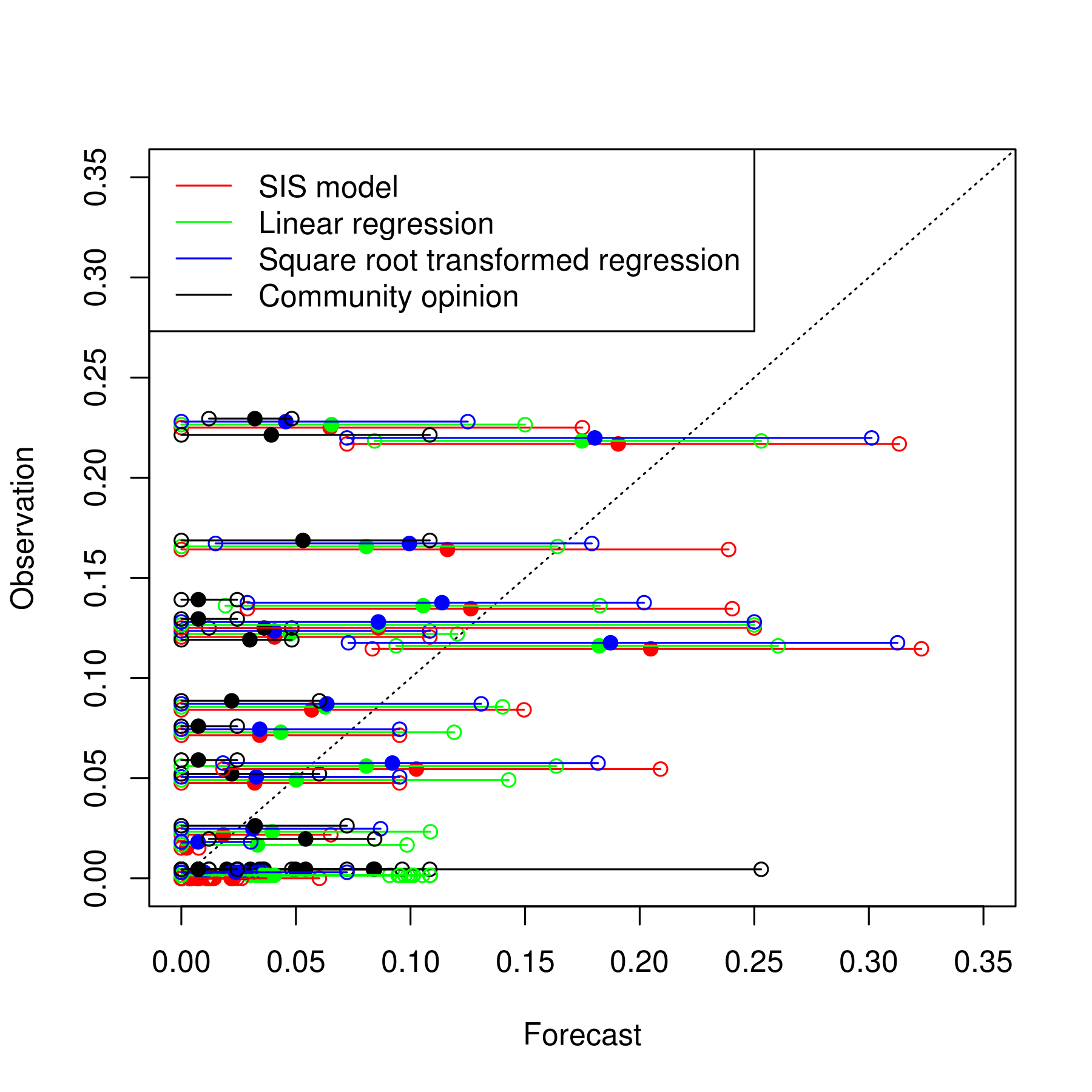

Supplement: S1 Fig — Regressions (linear regression, green; square root-transformed, blue), SIS hidden Markov Model (red), and community of experts (black), with mean (solid circle) and 95% CI (circle). (TIFF) [file pntd.0004000.s001.tiff]
