# Supplementary material for: Short-term Forecasting of the Prevalence of Trachoma: Expert Opinion, Statistical Regression, versus Transmission Models
Source: PLoS Negl Trop Dis. 2015 Aug 24;9(8):e0004000. doi: 10.1371/journal.pntd.0004000 (PMC4547743; doi:10.1371/journal.pntd.0004000)
Supplement: S1 Table — (DOCX) [file pntd.0004000.s002.docx]

**S1 Table. Difference between observed result and forecast by SIS, linear regression, square root transformed regression, or community opinion.**

|  | Difference the between observed prevalence and the mean of forecast prevalence | | | |
| --- | --- | --- | --- | --- |
| Village | SIS hidden Markov model | Linear regression | Square root regression | Community opinion |
| 1 | 0.080 | 0.073 | 0.080 | 0.084 |
| 2 | -0.007 | -0.035 | -0.007 | -0.020 |
| 3 | 0.003 | -0.018 | -0.010 | -0.010 |
| 4 | 0.160 | 0.159 | 0.179 | 0.193 |
| 5 | -0.004 | -0.030 | -0.010 | -0.030 |
| 6 | -0.004 | -0.035 | -0.009 | -0.034 |
| 7 | -0.007 | -0.033 | -0.007 | -0.084 |
| 8 | -0.008 | -0.038 | -0.008 | -0.054 |
| 9 | -0.003 | -0.034 | -0.009 | -0.050 |
| 10 | 0.013 | -0.018 | 0.008 | -0.039 |
| 11 | -0.007 | -0.039 | -0.007 | -0.036 |
| 12 | 0.048 | 0.083 | 0.065 | 0.111 |
| 13 | 0.016 | -0.003 | 0.015 | 0.026 |
| 14 | 0.027 | 0.021 | 0.021 | 0.062 |
| 15 | -0.090 | -0.068 | -0.073 | 0.085 |
| 16 | 0.026 | 0.042 | 0.036 | 0.178 |
| 17 | 0.039 | 0.039 | 0.039 | 0.118 |
| 18 | -0.003 | -0.035 | -0.009 | -0.007 |
| 19 | -0.004 | -0.035 | -0.010 | -0.007 |
| 20 | -0.048 | -0.026 | -0.038 | 0.047 |
| 21 | -0.004 | -0.037 | -0.009 | -0.007 |
| 22 | 0.008 | 0.029 | 0.021 | 0.127 |
| 23 | -0.022 | -0.041 | -0.024 | -0.007 |
| 24 | 0.037 | 0.028 | 0.037 | 0.064 |
